# Agent-based simulation of pedestrian dynamics for exposure time estimation in epidemic risk assessment

**DOI:** 10.1007/s10389-021-01489-y

**Published:** 2021-04-01

**Authors:** Thomas Harweg, Daniel Bachmann, Frank Weichert

**Affiliations:** Department of Computer Science, TU Dortmund University, 44227 Dortmund, Germany

**Keywords:** SARS-CoV-2, COVID-19, Pedestrian dynamics, Agent-based simulation, Social-force model, Numerical simulation

## Abstract

**Purpose:**

With the coronavirus disease 2019 (COVID-19) pandemic spreading across the world, protective measures for containing the virus are essential, especially as long as no vaccine or effective treatment is available. One important measure is the so-called *physical distancing* or *social distancing*.

**Methods:**

In this paper, we propose an agent-based numerical simulation of pedestrian dynamics in order to assess the behavior of pedestrians in public places in the context of contact transmission of infectious diseases like COVID-19, and to gather insights about exposure times and the overall effectiveness of distancing measures.

**Results:**

To abide by the minimum distance of 1.5 m stipulated by the German government at an infection rate of 2%, our simulation results suggest that a density of one person per 16m^2^ or below is sufficient.

**Conclusions:**

The results of this study give insight into how physical distancing as a protective measure can be carried out more efficiently to help reduce the spread of COVID-19.

## Introduction

Starting at the end of 2019, the coronavirus disease 2019 (COVID-19) was first described in Wuhan, China (Zhou et al. 2020), and rapidly spread world-wide over the past months causing an unprecedented pandemic with more than 431,541 deaths so far (first wave of disease) (World Health Organization 2020). The illness is caused by the severe acute respiratory syndrome coronavirus 2 (SARS-CoV-2). Despite drastic restrictions in everyday life, new infections are still on the rise (Dong et al. 2020). Hence, special attention should be given to the protection of vulnerable patients with high risk of a severe course of the disease. The diagnostic gold standard to identify SARS-CoV-2 infection is reverse transcription polymerase chain reaction (RT-PCR) of viral ribonucleic acid (RNA) collected by a combined nasopharyngeal swab (NPS) and oropharyngeal swab (OPS) (Zou et al. 2020). As long as no vaccine or at least no therapy is available, only exit restrictions and social distancing can slow the spread of COVID-19. Social distancing, also called “physical distancing” means limiting face-to-face contact with others. A model developed to support pandemic influenza planning (Ferguson et al. 2006; Halloran et al. 2008) was adapted using the data of the COVID-19 outbreak in Wuhan to explore scenarios for the United States and Great Britain (Ferguson et al. 2020) resulting in the advice of social distancing of the whole population and household quarantine of infected individuals as well as school and university closures. By simulating the COVID-19 outbreak in Wuhan using a deterministic stage-structured SEIR (susceptible, exposed, infectious, recovered) model over a 1-year period, Prem et al. (2020) came to the conclusion that a reduction in social mixing can be effective in reducing magnitude and delaying the peak of outbreak. These British epidemiologists from the London School of Hygiene & Tropical Medicine suspect that a second wave of COVID-19 disease could only be prevented if exit and contact restrictions were maintained over the long term or at least resumed intermittently. It is therefore increasingly important to know what distances must be maintained to avoid infection. As far as these distances are concerned, the study of Bischoff et al. (2013) revealed that healthcare professionals within 1.829 m of patients with influenza could be exposed to infectious doses of influenza virus, primarily in small-particle aerosols. This led to the advice of keeping a minimum distance of 1–2 m from the Robert Koch Institute (RKI) (Robert Koch Institute 2020a), which is evaluating available information of the corona virus and estimating the risk for the population in Germany. The RKI and the German Federal Ministry of Health has in response published a handout (Robert Koch Institute 2020b) stating that in public, a minimum distance of 1.5 m must be maintained wherever possible. Besides, it is relevant whether people are next to each other, staggered behind each other, or directly behind each other. Especially in indoor areas (e.g., shopping), corresponding constellations occur in combination. Simulations can help to make the necessary distance measures easier to understand. Different distance scenarios can be simulated and recommendations for the distance can be suggested.

In their call to action, (Squazzoni et al. 2020) give an overview of computational models for global pandemic outbreak simulation and their limitations and Chang et al. (2020) propose a microscopic model for the simulation of the COVID-19 outbreak in Australia. The model consists of over 24 million individuals with different characteristics and social context and was calibrated with the 2016 census data of Australia.

In the context of the simulation of pedestrian dynamics, there exists a variety of comprehensive surveys (Shiwakoti et al. 2008; Schadschneider et al. 2009; Caramuta et al. 2017). Models were differentiated mainly into macroscopic and microscopic. In macroscopic models, the crowd is assumed as the smallest entity. These models allow the representation of high-density crowds, but cannot model the interaction between pairs or groups of pedestrians. Microscopic models assume one pedestrian as the smallest entity. They are roughly divided into physical force models, cellular automata, or queueing models. This work focusses on the so-called social force model by Helbing (1991, 2000, 2001, 2002) which can be categorized as a physical force model.

The main contributions of this work are

- adaptable social force-based model for pedestrian dynamics in realistic environments
- exposure time measurement for assessment of the spreading of diseases
- discussion of the effects of distancing measures on exposure times.

This paper is organized as follows. Firstly, “Related work” gives an overview of existing models for simulations of the spread of diseases. In “Materials and methods”, we set forth the agent-based model and the accompanying simulation we used for our experiments. “Experiments” describes the experiments concerning the simulation of pedestrian dynamics in realistic environments, the effectiveness of distancing measures, and measuring of exposure time in the context of infectious diseases in general and COVID-19 in particular. Following, in “Results”, we present the results from the experiment, and in “Discussion” we give a short conclusion/summary and outlook.

## Related work

As far as the simulation of the spreading of diseases is concerned, most approaches are based on macroscopic models. The so-called compartment models (Brauer 2008) divide the population of interest into compartments with different characteristics. The simplest model is the SIR model. It consists of three compartments *susceptible*, *infectious*, and *recovered*. The population is split into these three compartments. Entities in the susceptible group model the entities most likely to be infected. The entities in the infectious group are the ones already infected, and the entities in the recovered group have recovered from the infection. An entity transfer from for example susceptible to infectious state and from infectious to recovered state could be modeled. Thus, re-infection with the modeled disease would not be depicted by this model. A plethora of different variations of this scheme exist with varying numbers of compartments (Hethcote 2000). The independent variable in the compartment models is time *t*. The transfer ratios of the population from one compartment to another are expressed as derivatives with respect to *t*, thus resulting in differential equations for the compartments of the model. One of the shortcomings of these models is that each individual in the population is modeled with the same set of features. This is overcome by the introduction of metapopulations (Brockmann et al. 2006; Balcan et al. 2009) building sub-compartments of, e. g., entities with natural immunity or asymptomatic individuals. Still, each of the metapopulations share a homogeneous set of model parameters.

With the rapid growth of available processing power, individual-based models (IBM) or agent-based models (ABM) are used to model infectious disease outbreaks. Willem et al. (2017) and Nepomuceno et al. (2019) give comprehensive overviews of IBM/ABM usage in the field of epidemiology. Based on the work by Brockmann (2010), Frias-Martinez et al. (2011) propose an agent-based model of epidemic spread based on social network information from data of base transceiver stations (BTC) captured during the 2009 H1N1 outbreak in Mexico. By simulating an outbreak of measles that occurred in Schull, Ireland, in 2012 based on open data, Hunter et al. (2018) have shown recently, that agent-based modeling in combination with open data leads to regionally transferable models. Bobashev et al. (2007) discuss the combination of compartment models and microscopic agent-based models into a so-called hybrid multi-scale model.

In this paper, we propose a microscopic model for simulating pedestrian dynamics in the context of infectious disease spread, including monitoring of contacts and exposure times in realistic scenarios. The simulation devised in this work runs in real-time, giving instantaneous feedback about the considered scenario and thus allowing for visual assessment, in addition to resulting statistics.

## Materials and methods

In the following, we describe in detail the underlying physical model of pedestrian dynamics and the simulation we implemented. Our simulation is adapted to realistic scenarios in the context of the assessment of infectious disease spread, with focus on exposure time measurement. Specifically, we apply it to a supermarket scenario and the corresponding measures taken with regard to the COVID-19 pandemic.

The simulation we present is an agent-based approach based on the model of Helbing(1991, 2000, 2001, 2002). The simulation is carried out on a static scenery with a defined number $n \in \mathbb {N}$ of *agents* or *particlesp*_*i*_. Each agent has an individual starting point **s**_*i*_ and destination point **d**_*i*_(*t*) (both $\in \mathbb {R}^{2}$), the former being static and the latter varying with time $t \in \mathbb {R}_{\geq 0}$.

Motion of an individual *p*_*i*_ is governed by Eq. 1, which is composed of the term for self-propelling **a**^self^ and external forces **f** acting upon *p*_*i*_

1 $$  \frac{d\mathbf{x}^{2}_{i}(t)}{dt^{2}} = \mathbf{a}^{\text{self}}_{i} + \frac{1}{m_{i}} \cdot \left( \sum\limits_{j} (\mathbf{f}_{ij}^{\text{soc}} + \mathbf{f}_{ij}^{ph}) + \mathbf{f}^{\text{wall}}_{i})\right)\textrm{.} $$

Here, $\mathbf {x}_{i}\in \mathbb {R}^{2}$ denotes the position of *p*_*i*_, and *m*_*i*_ (in kg) its mass.

Self-acceleration of agents, as defined in Eq. 2, is the adjustment of the actual velocity **v**_*i*_(*t*) to the desired velocity

2 $$  \mathbf{a}^{\text{self}}_{i}(t) = \frac{{v^{0}_{i}}\mathbf{e}^{0}_{i}(t) - \mathbf{v}_{i}(t)}{\tau}\textrm{.} $$

The parameter $\tau \in \mathbb {R}_{>0}$ (in s) determines the amount of delay time for an agent to adapt. The desired velocity of an agent *p*_*i*_ is the product of its speed ${v^{0}_{i}}$ and the desired direction of movement $\mathbf {e}^{0}_{i}(t)$. The latter depends on the destination **d**_*i*_(*t*) and is determined by the pre-computed navigation method described below.

The model by Helbing mainly consists of different types of forces acting upon an agent, social and physical forces. The social forces **f**^soc^ model a pedestrian’s endeavor to avoid contact with other pedestrians, while the physical forces **f**^ph^ model effects arising when pedestrians are so close that there is actual contact between them. While in the original model of Helbing et al. the social force is only comprised of a normal component (called **f**^norm^ here), in this paper, we add an additional tangential term **f**^tang^.

The social force thus takes the following form:

3 $$  \mathbf{f}^{\text{soc}} = \mathbf{f}^{\text{norm}} + \mathbf{f}^{\text{tang}}. $$

**Fig. 1:**
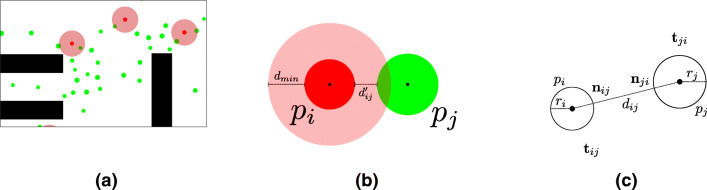
Agent interaction in the simulation. (**a**) Visualization of the running simulation. (**b**) The measuring of distances. (**c**) The associated quantities of the underlying physical model

The accompanying force in normal direction is defined as usual (Helbing 1991, 2002):

4 $$  \mathbf{f}^{\text{norm}}_{ij}(t) = A \cdot \exp \left( \frac{r_{ij}-d_{ij}(t)}{B} \right) \cdot \mathbf{n}_{ij}(t)\cdot \left( \lambda+(1-\lambda)\frac{1+\cos\phi_{ij}(t)}{2}\right). $$

where *A* determines the force (in N) and *B* the range (in m) of repulsive interactions, and *λ* ∈ [0,1] the (an-)isotropy. The vector **n**_*ij*_(*t*) denotes the normal from *p*_*i*_ to *p*_*j*_ of unit length, *ϕ* the angle between current direction **e**_*i*_(*t*) of particle *p*_*i*_ and **n**_*ij*_(*t*). The scalar value *r*_*ij*_ is the sum of the respective radii *r*_*i*_ and *r*_*j*_ of the considered particles, while $d_{ij}(t) = \lVert \mathbf {x}_{i}(t)-\mathbf {x}_{j}(t)\rVert $ is the distance between their centers, both quantities are measured in meters. Note that for the experiments performed, the distance *dij*′ between the perimeters is measured. This is defined as $d^{\prime }_{ij} = d_{ij}-r_{ij}$. With regard to the measures taken by the German government for prevention of the spread of COVID-19, we set the critical distance *d*_*min*_ for a possible transmission to 1.5 m. Figure 1 shows different aspects of agent interaction. Figure 1a and b show interaction in the simulation with regard to the critical distance, while Fig. 1c shows associated quantities of the mathematical model between two particles.

The tangential term **f**^tang^ (5) is now defined as a fraction $\gamma \in \mathbb {R}$ of the normal term, but in direction **t**_*ij*_, orthogonal to **n**_*ij*_. Furthermore, this term is added only if the pedestrians involved, *p*_*i*_ and *p*_*j*_, are heading into opposite directions. This is taken care of by the function *ψ*_*ij*_ (6).

5 $$  \mathbf{f}^{\text{tang}}(t) = \psi_{ij}(t) \cdot \gamma \cdot \lVert\mathbf{f}^{\text{norm}}_{ij}(t)\rVert \cdot \mathbf{t}_{ij}(t). $$

The value of *γ* ∈ [0,1] determines the amount of normal social force added in tangential direction. For the experiments performed, we set *γ* = 0.7. The function *ψ* (6) determines the pedestrians’ directions by evaluating the dot product between their respective directions of movement $\mathbf {e}_{i}(t) = \frac {\mathbf {v}_{i}(t)}{\lVert \mathbf {v}_{i}(t)\rVert }$:

6 $$  \psi_{ij}(t) = \begin{cases} 1 & \langle\mathbf{e}_{i}(t), \mathbf{e}_{j}(t)\rangle \leq 0 \\ 0 &  \text{otherwise}\textrm{.}\\ \end{cases} $$

The added tangential term makes for a more realistic movement of the agents, as they evade each other early, if applied. This is even more noticeable in conjunction with the method we employ for pathfinding, as described below.

Physical forces acting between pedestrians are also defined in conformance to Helbing et al:

7 $$  \mathbf{f}^{\text{ph}}_{ij}(t) = k \cdot {\varTheta}(r_{ij}-d_{ij}(t)) \cdot \mathbf{n}_{ij}(t) + \kappa \cdot {\varTheta}(r_{ij}-d_{ij}(t)) \cdot {\Delta} v^{t}_{ji}(t) \cdot \mathbf{t}_{ij}(t). $$

Here, constants *κ* and *k* determine amounts of friction and the force counteracting body compression. The term ${\Delta } v^{t}_{ji} = (\mathbf {v}_{j} - \mathbf {v}_{i}) \cdot \mathbf {t}_{ij}$ describes the tangential speed difference between agents *p*_*i*_ and *p*_*j*_. The function *Θ* ensures that physical forces only arise when two particles actually touch, i. e. the distance *d*_*ij*_ between them is lower than the sum of their radii *r*_*ij*_:

8 $$  {\varTheta}(x) = \begin{cases} 0 & \text{if } x < 0 \\ x & \text{otherwise} \textrm{.}\end{cases} $$

Finally, wall forces **f**^wall^ define interaction of agents with obstacles, like walls and stationary objects. The wall forces are defined in analogy to particle forces:

9 $$ \begin{array}{@{}rcl@{}} \mathbf{f}^{\text{wall}}_{i}(t) &=& \mathbf{f}^{\text{wsoc}}_{i}(t) + \mathbf{f}^{\text{wph}}_{i}(t) \\ &=& \left( A^{\text{wall}} \cdot \exp\left( \frac{r_{i}-d_{ib}(t)}{B^{\text{wall}}}\right) + k \cdot {\varTheta}(r_{i}-d_{ib}(t))\right) \cdot \mathbf{n}_{ib}(t) \\ &-& \kappa \cdot {\varTheta}(r_{i}-d_{ib}(t)) \cdot (\mathbf{v}_{i}(t)\cdot\mathbf{t}_{ib}(t)) \cdot \mathbf{t}_{ib}(t). \end{array} $$

In Eq. 9, *d*_*ib*_ denotes the distance, and **n**_*ib*_ the normal and **t**_*ib*_ the tangential direction towards the closest obstacle(-point) *b* to *p*_*i*_, which are all determined directly from the scene representation described below. Parameters *A*^wall^ and *B*^wall^ are the analogues to *A* and *B* in Eq. 4. This is a slight deviation from the model by Helbing et al., as we define separate social distance parameters for obstacles and for other agents.

**Fig. 2:**
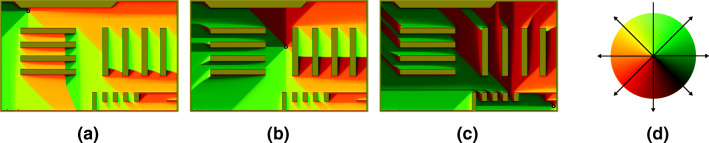
Color-coded direction maps for agent navigation, determining directions of movement for each position within walkable areas (**a–c**). Destinations are shown as *black discs with white dots*.(**d**) How colors are mapped to directions

**Fig. 3:**
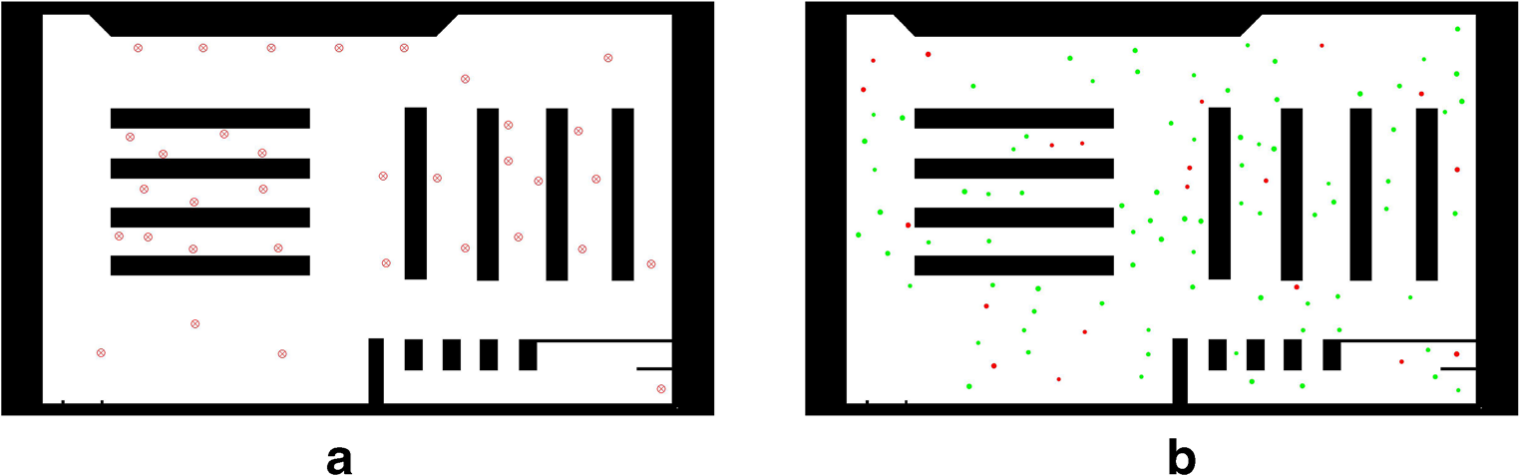
Supermarket scene with walkable areas shown in *white* and obstacles in *black*

For the experiments conducted in this work, a scene the agents can move around in needs to be defined. This will define the supermarket scenario considered in the simulation we performed with regard to the COVID-19 measures, as described in “Experiments”. The scene is represented as a distance transform ((Borgefors 1986; Felzenszwalb and Huttenlocher 2004)) of a two-dimensional, binary discretized map, as depicted in Fig. 3. This map defines the size of the simulated area, as well as the regions within which are walkable (shown as white) or pose an obstacle to the agents (black). The scene in the experiments performed is represented by a discretized map at a resolution of 8 pixels per meter. Agent navigation depends on pre-calculated paths based on *Dijkstra’s* algorithm ((Dijkstra 1959)). Accordingly, for each destination, a map containing the directions of movement towards it for each walkable point in the area is generated. This way, the direction of movement **e**_*i*_(*t*) of an agent *p*_*i*_ is determined by looking up the given direction of movement at position **x**_*i*_ from the map corresponding to *p*_*i*_’s current destination **d**_*i*_ (cf.“Materials and methods”). Color-coded renderings of maps for three different destination points are shown in Fig. 2. Agent navigation in the context of our COVID-19 simulation, including details on how destinations are chosen, is described in “Experiments”.

The need for the added tangential social force **f**^tang^ becomes apparent in cases of symmetry, which arise when forces are at an equilibrium. The pre-calculated paths may impose symmetries, as they minimize the distance towards the destination. This means, the prescribed way can be a thin line even though there is more space available, resulting in unnatural behavior and building of queues, especially when agents are approaching others head-on. In the worst case, this can lead to “deadlocks” or clogging of pedestrians, even in cases where the surrounding area provides enough space for the agents to evade each other.

## Experiments

The simulation was carried out with a basic “supermarket” scenario of size 80m × 60m (cf. Fig. 3) with varying numbers *n* of agents, which represent customers. The scene is aimed to mimic a typical German supermarket with shelves, counters, and cashiers. Within the simulated area, a set of 34 destinations is defined (Fig. 3a), representing points of interest within the supermarket. We performed simulations for number of agents (population size) of *n* ∈{50,100,200,300}. A defined amount of the agents were marked as “infected”.

Figure 3b shows the scene with 100 agents. Infectious persons are marked in red in the visualization, and all others are in green. Infected agents carry the virus and can potentially infect others. The amount of infected agents was varied from {0.02,0.05,0.1,0.15,0.2} in the experiments, tantamount to ratios of 2*%* to 20*%*. The agents’ radii *r*_*i*_ were sampled uniformly from [0.25,0.35]. Mass is then determined proportionally to the individual radii as *m*_*i*_ = 160 ⋅ *r*_*i*_ in kg. The desired speed ${v_{i}^{0}}$ was sampled uniformly from the range [0.3,0.8] (in ms).

Simulation parameters were chosen as *A* = 10000N, *B* ∈{0.5,0.7,1.0,1.5}m, *A*^wall^ = 10000N, *B*^wall^ = 0.5m, and *τ* = 0.5s. As body contact hardly ever occurs, constants *k* and *κ* supposedly have very little impact on the outcome of the simulation, if at all. For the sake of completeness, we set *k* = 20000kg ⋅m^− 1^ ⋅s^− 1^, and *κ* = 40000kg ⋅s^− 2^.

Initially, agents were distributed randomly across the area, i. e. their starting points **s**_*i*_ are set to random (walkable) positions within the scene. The set of destinations were assigned to the agents in an even split, determining the agents’ initial destination points **d**_*i*_. During the simulation, if an agent reaches its destination, a new destination is assigned randomly from the available set of destinations (excluding the current one). Agent movement is then determined by the direction map corresponding to the destination, as described in “Materials and methods”. Thus, a typical behavior of customers walking around the supermarket is simulated. The simulation time was set to 15 min.

During the simulation, for each agent, we keep track of exposure time to infected agents. More precisely, the time span an agent comes below the prescribed safety distance *d*_*min*_ of 1.5m (cf. “Introduction”) to an infected agent is accumulated per individual during the course of the simulation.

## Results

**Fig. 4:**
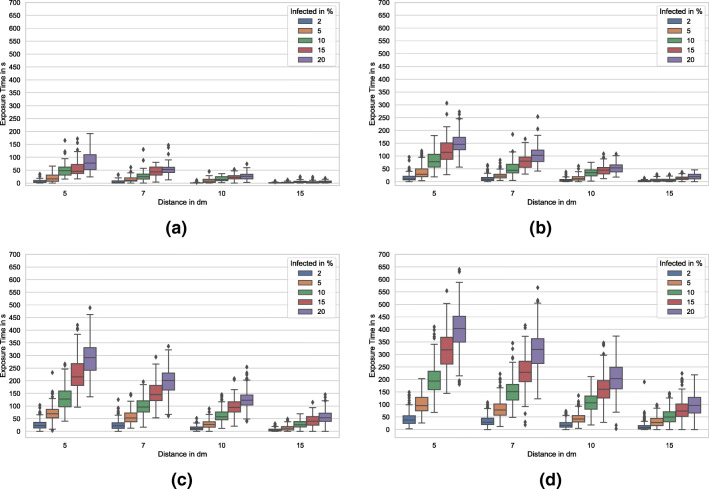
Box-and-whisker plots of the exposure times for populations of *n* ∈{50,100,200,300}, desired distances of 50 cm, 70 cm, 1 m, and 1.5 m and infection rates of 2, 5, 10, 15, and 20%

The box-and-whisker plots (Tukey 1970) in Fig. 4 show the average deviation of the exposure time concerning the different population sizes, distances, and infection rates, as described in “Experiments”. The boxes represent the interquartile range, which contains 50% of the values and the whiskers are marking the minimum and maximum values, excluding outliers (marked as black diamonds).

The first scenario depicted in Fig. 4a shows the results for a minimal population of 50 agents. With a uniform distribution of the individuals and if superstructures (cf. Fig. 3a) are neglected a density of one individual per 96m^2^ is to be expected. Thus, enough space for avoidance is available. This is supported by the box plots. Even if a very short desired distance of 50 cm is considered, the mean exposure time is 7.78s (standard deviation (SD) 9.1s) for an infection rate of 2% of the individuals. Even if very high infection rates of 10% are considered the mean, exposure time is below 1 m (52s, SD 29.16s). If the requested distance of 1.5m (cf. “Introduction”) is maintained, mean exposure time for an infection rate of 20% is 2.3s (SD 2.9s). With the growing number of agents, exposure times are rising (cf. Fig. 4d). Expected density with a population of 100 is one individual per 48m^2^. For small desired distances and medium infection rates of 5%, the mean exposure time is 84.95s (SD 36.5s), which is 61% higher than the results with the same parameters and a population of 50. As the desired distances increase, the exposure times are decreasing. Considering the requested 1.5-m distance, the mean exposure time for an infection rate of 20% is 19.85s (SD 11.79s), which is 836% higher than the exposure times for a population of 50. As far as realistic infection rates of 2% are concerned, the mean exposure time is 2.49s (SD 2.84s). The results for a population size of 200 are shown in Fig. 4c. This population size with an expected density of one agent per 24m^2^ representing the maximum density allowed during lock down in most of German federal states at the time of writing. For an infection rate of 2% and a desired distance of 1.5m, the mean exposure time is 6.19s (SD 6.02s). With an expected density of one individual per 16m^2^, Fig. 4d shows the results for a simulation with 300 agents. Mean exposure time is 11.31s (SD 15.03s). The 50% percentile (median) for all simulations with this parametrization is below 8s (7.7s, 4.64s, 1.37s, 0s), showing the effectiveness of distancing tactics in the minimization of exposure times.

## Discussion

We have presented a simulation of pedestrian dynamics in realistic scenarios with a focus on the spread of infectious diseases by contact transmission. An important measure taken to reduce the spread of COVID-19 is the so-called *social distancing* or *physical distancing*, aiming to reduce close contacts between individuals in public places. In the experiments we conducted, we showed how our simulation can give insights about exposure time to infected individuals and the feasibility and effectiveness of keeping distance in realistic crowded scenarios. Our experiments suggest that, if we assume an infection rate of 2%, the prescribed minimum distance of 1.5m can be maintained if a density of one person per 16m^2^ is not exceeded.

In this work, we have presented a method for risk assessment concerning pedestrian dynamics and exposure time in conjunction with COVID-19 in particular and infectious diseases in general. Due to the flexibility of the approach, it can be applied to a great variety of scenarios prone to transmission of contagious diseases. This especially includes public places, indoor as well as outdoor.

Our simulation can serve as a tool for a better assessment of quantities regarding the number of people to admit, or on guidelines for distances to keep between individual persons. Due to the nature of the simulation, it can also give insight about optimization on the geometry of the surrounding, like identification of bottlenecks and hotspots, in order to reduce risks for people moving around and meeting in the place in question.

It should be noted that there may be further factors to be taken into consideration which we do not cover in the current version of our simulation. Especially for indoor environments, ventilation, air flow, and corresponding aspects of fluid dynamics might be of importance for more complete assessment of the risk of virus transmission (Kumar and Morawska 2019; Morawska and Milton 2020).

There already are studies on the topic of indoor ventilation (Licina et al. 2015; Jung et al. 2015; Nielsen 2009), also with focus on COVID-19 (Morawska et al. 2020) and fluid flow in particular (Bhagat et al. 2020). Integration of approaches like these into the simulation we presented could make for a more accurate risk assessment of public spaces. This constitutes a promising subject for future research.

With the COVID-19 pandemic affecting countries all over the world at the time of writing, the urgent need of models and tools for better assessment of situations in public places is apparent. We are confident that our simulation results can serve as a basis for better risk assessment in public places in the context of infectious diseases, and for further research in this area.

## Data Availability

Will be made available upon request.

## Code Availability

Will be made available upon request.

## Declarations

Research performed in this study did not involve any human participants or animals.
